# O19 - Changes in the balance between myeloid (mDC) and plasmacytoid (pDC) cell numbers in peripheral blood during childhood

**DOI:** 10.1186/2045-7022-4-S1-O19

**Published:** 2014-03-14

**Authors:** Paraskevi Maggina, Vassiliki Papaevangelou, Maria Tsolia, Nikolaos G Papadopoulos

**Affiliations:** 1 2nd Department of Pediatrics, “P. and A. Kyriakou” Children's Hospital, National and Kapodestrian University of Athens, Athens, Greece

## Background

Dendritic cells (DCs) are the most potent antigen-presenting cells, having an important role in linking innate and adaptive immunity. DCs are also critical mediators of immune tolerance and anergy, depending on the type of antigen they encounter. Peripheral blood DCs represent only the 0.1–1% of mononuclear cells and, based on their lineage origins, they can be divided into two major subsets, plasmacytoid DCs (pDCs) and myeloid DCs (mDCs). Although, the total number of all blood circulating lymphocyte subpopulations in children declines with age, previous studies performed on age-related DC changes have shown controversial results [1-4].

## Material and methods

Blood samples were obtained from 43 clinically healthy children aged between 1 day to 11 years old during routine examinations for minor elective surgery or routine checkups in the outpatient clinic. DCs were identified, by FACScan flow cytometer, as showing no labeling for the “lineage cocktail” (fluorescein isothiocyanate (FITC)-conjugated monoclonal antibodies including CD3, CD14, CD16, CD19, CD20, CD56) and strong labeling for HLA-DR. The percentages of mDCs (CD 11+) and pDCs (CD 123+) were determined using three-colour flow cytometry and their absolute numbers were calculated by using their percentage in relation to the lymphocyte and monocyte number, as determined by differential blood count.

## Results

Similarly to previous studies’ findings [2,3], we demonstrate that while mDCs do not change with age, pDCs decrease significantly with age (linear regression: p=0.0242, R^2^=0.1343). Moreover, the mDCs/pDCs ratio showed a significant positive correlation with age during childhood (linear regression: p=0.0044, R^2^=0.2092).

## Conclusion

The human immune system is functional less mature during infancy and within the first years of life. Although young children show adult levels of mDCs, the dynamic changes in the balance between mDCs and pDCs during childhood may play a role in the vulnerability of young children to viral and bacterial infections.
